# Phosphorylation Regulates OLIG2 Cofactor Choice and the Motor Neuron-Oligodendrocyte Fate Switch

**DOI:** 10.1016/j.neuron.2011.01.030

**Published:** 2011-03-10

**Authors:** Huiliang Li, Joana Paes de Faria, Paul Andrew, Justyna Nitarska, William D. Richardson

**Affiliations:** 1 Wolfson Institute for Biomedical Research and Research Department of Cell and Developmental Biology, University College London, Gower Street, London WC1E 6BT, UK; 2 MRC Laboratory for Molecular Cell Biology, University College London, Gower Street, London WC1E 6BT, UK

## Abstract

A fundamental feature of central nervous system development is that neurons are generated before glia. In the embryonic spinal cord, for example, a group of neuroepithelial stem cells (NSCs) generates motor neurons (MNs), before switching abruptly to oligodendrocyte precursors (OLPs). We asked how transcription factor OLIG2 participates in this MN-OLP fate switch. We found that Serine 147 in the helix-loop-helix domain of OLIG2 was phosphorylated during MN production and dephosphorylated at the onset of OLP genesis. Mutating Serine 147 to Alanine (S147A) abolished MN production without preventing OLP production in transgenic mice, chicks, or cultured P19 cells. We conclude that S147 phosphorylation, possibly by protein kinase A, is required for MN but not OLP genesis and propose that dephosphorylation triggers the MN-OLP switch. Wild-type OLIG2 forms stable homodimers, whereas mutant (unphosphorylated) OLIG2^S147A^ prefers to form heterodimers with Neurogenin 2 or other bHLH partners, suggesting a molecular basis for the switch.

## Highlights

► OLIG2(S147) dephosphorylation precedes the motor neuron (MN)-oligodendrocyte (OL) fate switch ► Mutant OLIG2(S147A) can induce OL but not MN fate ► Dephosphorylated S147 favors OLIG2-NGN2 over OLIG2-OLIG2 dimers ► This suggests a sequestration model for the MN-OL fate switch

## Introduction

All the neurons and glial cells of the mature central nervous system (CNS) are generated by neuroepithelial stem cells (NSCs) in the ventricular zone (VZ) that surrounds the lumen of the embryonic neural tube (forerunner of the spinal cord and brain). Cell diversification occurs in stages. First, a neurogenic prepattern is laid down in the plane of the VZ, under the action of graded morphogens released from organizing centers within or outside the neural tube. This leads to a mosaic of molecularly distinct progenitor domains, each of which goes on to generate a characteristic subset of neurons and glia. Superimposed on this spatial pattern is a temporal pattern of cell generation from some regions of the VZ. For example, in the developing cerebral cortex, different classes of projection neuron are generated in sequence (Shen et al., 2006); these settle in stereotypic positions to generate the layered structure of the cortex. Subsequently, cortical NSCs start to produce glial lineages (astrocytes and oligodendrocytes [OLs]). This late neuron-glial switch is a general property of NSCs in all parts of the developing brain and spinal cord. In some areas of the VZ, NSCs switch from neuron to astrocyte production, whereas other regions generate oligodendrocyte precursors (OLPs), which migrate widely before differentiating into myelin-forming OLs (Rowitch, 2004; Richardson et al., 2006). Less is known about the temporal control of cell fate than the spatial patterning that precedes it. We set out to study this temporal aspect of cell diversification, focusing on neuron-glial switching in the ventral spinal cord.

Spatial pattern in the ventral half of the developing spinal cord is established largely through the action of Sonic hedgehog (SHH) protein released from the notochord and floor plate at the ventral midline. SHH activates or inhibits different sets of transcription factors at different distances from the floor plate (different concentrations of SHH). Subsequently, cross-repressive interactions among the transcription factors expressed in adjacent regions of the VZ establish sharp boundaries of gene expression in the dorsal-ventral axis, establishing a set of ribbon-like NSC domains that run parallel to one another along the neuraxis. In the ventral half of the cord, these domains are known (from ventral to dorsal) as p3, pMN, p2, p1, and p0 (Jessell, 2000). Six additional NSC domains (dP1–dP6, dorsal to ventral) are formed in the dorsal half of the spinal cord under the influence of BMPs and WNTs secreted from the roof plate (Helms and Johnson, 2003). NSCs in the ventral pMN domain generate several different subtypes of motor neuron (MN) before switching abruptly to OLP production (reviewed by Richardson et al., 2000). NSCs in the neighboring p3 and p2 domains generate interneurons followed by astrocytes (Rowitch et al., 2002).

The pMN domain contributes all of the MNs and ∼80% of the OLPs in the mouse spinal cord (Fogarty, 2006; Richardson et al., 2006). The remaining OLPs are generated outside pMN in a SHH-independent manner (Cai et al., 2005; Fogarty et al., 2005; Vallstedt et al., 2005). pMN is marked by transcription factor OLIG2 and its close relative OLIG1, which were originally identified in screens for OL-specific genes (Lu et al., 2000; Takebayashi et al., 2000; Zhou et al., 2000). OLIG2 knockout results in loss of the pMN domain and consequently complete absence of spinal MNs (Lu et al., 2002; Takebayashi et al., 2002; Zhou and Anderson, 2002; Park et al., 2002). All spinal OL lineage cells are lost as well because OLIG2 is required for OLP development regardless of whether they are generated within or outside of pMN (Lu et al., 2002; Takebayashi et al., 2002; Zhou and Anderson, 2002; Park et al., 2002). In contrast, OLIG1 has a relatively mild impact on normal development (Lu et al., 2002; J.P.d.F., N. Kessaris, W.D.R., and H.L., unpublished data; but see Xin et al., 2005). However, OLIG1 is believed to be crucial for OL regeneration in demyelinating diseases such as multiple sclerosis (Arnett et al., 2004).

The OLIG gene products are members of a large family of helix-loop-helix (HLH) transcription factors, which also includes proneural proteins Neurogenin1/2 (NGN1/2) and MASH1/ASCL1 as well as cell lineage regulators MYOD and NEUROD. OLIG2 interacts with different protein partners to regulate specific developmental processes. It can form heterodimers with NGN2 to control MN differentiation, and it can bind NKX2.2 to promote OLP generation and/or differentiation (Novitch et al., 2001; Zhou et al., 2001; Qi et al., 2001; Sun et al., 2003; Lee et al., 2005). It can also complex with SOX10 or ZFP488 to regulate OLP differentiation and enhance myelin gene expression (Wang et al., 2006; Wissmuller et al., 2006; Li et al., 2007).

Given the central role of OLIG2 in both MN and OL development, we were keen to discover how this one transcription factor can specify two quite different cell types and especially how it participates in the MN-OLP temporal fate switch. We present evidence that OLIG2 controls the switch by reversible phosphorylation on Serine 147 (S147), a predicted protein kinase A (PKA) target; phosphorylation at this site is required for patterning of the ventral neuroepithelium and MN specification, whereas dephosphorylation favors OLP specification. S147 phosphorylation also causes OLIG2 to switch its preferred dimerization partner from OLIG2 (or OLIG1) to NGN2. We propose that this regulated exchange of cofactors is required for and triggers the MN-OLP fate switch.

## Results

### OLIG2 Is Reversibly Phosphorylated on Serine 147

**Figure 1:**
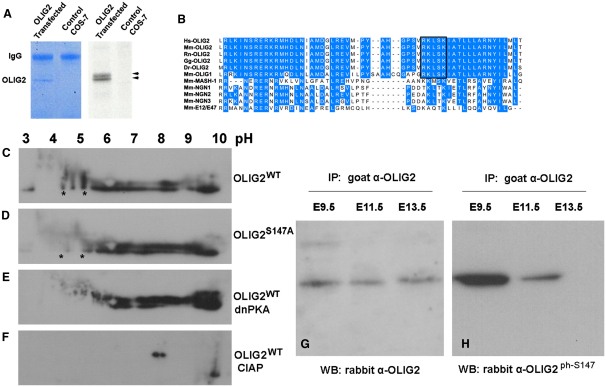
OLIG2 Is Phosphorylated on S147 (A) In vitro [^33^P] incorporation assay. Cell lysates of *Olig2*-transfected Cos-7 cells were immunoprecipitated with rabbit anti-Myc, separated by SDS-PAGE, and stained with Coomassie blue (left panel). The gel was dried and autoradiographed (right panel). OLIG2 bands are indicated (arrowheads). (B) Sequence alignment of bHLH domain of OLIG2 and its relatives. A predicted PKA phosphorylation motif containing S147 (black box) was found in the OLIG1/2 bHLH domain (helix 2) of different species. Hs, *Homo sapiens*; Mm, *Mus musculus*; Rn, *Rattus norvegicus* (brown rat); Gg, *Gallus gallus* (chicken); Dr, *Danio rerio* (zebrafish). (C–F) Transfected Cos-7 cells were analyzed by 2D PAGE followed by WB with anti-Myc antibody: (C) Myc-tagged OLIG2^WT^; (D) OLIG2^S147A^; (E) OLIG2^WT^ with dnPKA; and (F) OLIG2^WT^ lysate treated with CIAP. (G and H) OLIG2-S147 was phosphorylated in vivo. Mouse spinal cord homogenates from different developmental stages were analyzed by IP with goat anti-OLIG2, followed by WB with (G) rabbit anti-OLIG2 or (H) rabbit anti-OLIG2 ^ph-S147^. See also Figure S1 and Table S1.

OLIG2 is rich in serine and threonine residues (50 serines and 14 threonines out of a total of 323 amino acids; see Table S1 available online), suggesting that it might possess multiple serine/threonine phosphorylation sites. To test this, we transfected a Myc epitope-tagged OLIG2 expression vector into Cos-7 cells, labeled the cells with [^33^P]phosphate, and analyzed radiolabeled OLIG2 proteins by immunoprecipitation (IP) with anti-Myc followed by polyacrylamide gel electrophoresis (PAGE). Two major radioactive OLIG2 bands were visible (Figure 1A). Separation of *Olig2*-transfected Cos-7 cell lysates by two-dimensional (2D) PAGE (pH gradient in the second dimension) followed by Western blotting (WB) confirmed that OLIG2 was phosphorylated on multiple sites and that most of the phosphates could be removed by calf intestinal alkaline phosphatase (CIAP) (Figures 1C and 1F). Bioinformatic analysis of OLIG2 using online services NetPhos 2.0 Server (Blom et al., 1999) and NetPhosK 1.0 Server (Blom et al., 2004) identified one potential PKA and one potential protein kinase C (PKC) phosphorylation site (Table S1). Alignment of OLIG2 protein sequences from different species revealed that the predicted PKA site (R[R/K]X[S/T]) (X, any amino acid)—at S147 in the bHLH Helix-2 (H2) domain—is conserved during evolution (Figure 1B). This site is also conserved in OLIG1 but not in NGN1–3 or other bHLH proteins examined. We mutated S147 to Alanine (S147A), transfected the mutant (*Olig2^S147A^*) and wild-type (*Olig2^WT^*) constructs into Cos-7 cells, and visualized OLIG2 proteins by 2D PAGE and WB as before. This revealed an altered phosphorylation pattern of OLIG2^S147A^ (Figure 1D), indicating that S147 is a bone fide phosphate acceptor. Moreover, cotransfection of *Olig2^WT^* with a plasmid encoding a dominant-negative form of PKA (dnPKA) strongly reduced the phosphoprotein signal in the low pH range of the 2D gel (the region affected by S147A mutation), implying that OLIG2 can be phosphorylated by PKA on several sites, including S147 (Figure 1E).

### OLIG2-S147 Is Phosphorylated In Vivo during MN Development

To examine OLIG2-S147 phosphorylation status directly, we raised an antiserum in rabbits against a custom phosphopeptide and purified an antibody fraction that specifically recognizes the S147-phosphorylated form of OLIG2 (anti-OLIG2 ^ph-S147^; Figure S1). We prepared nuclear extracts of embryonic day 9.5 (E9.5), E11.5, and E13.5 mouse spinal cord tissue, immunoprecipitated endogenous OLIG2 protein with a goat anti-OLIG2 antibody, and visualized the precipitates by PAGE and WB with either rabbit anti-OLIG2 or rabbit anti-OLIG2 ^ph-S147^. OLIG2 was expressed more or less equally at all stages examined (Figure 1G). The specific phosphorylated form OLIG2 ^ph-S147^ was present at E9.5 and to a lesser extent at E11.5 (∼3.2-fold decrease; see Experimental Procedures) but was not detected at E13.5 (Figure 1H). These results demonstrate that endogenous OLIG2 is phosphorylated on S147 during MN production (∼E9–12) but is later dephosphorylated, coinciding with the switch from MN to OLP production that occurs around E12.5 in mice (Pringle et al., 1996; Richardson et al., 2006).

### Mutation of S147 to Alanine Changes the Protein Binding Properties of OLIG2

**Figure 2:**
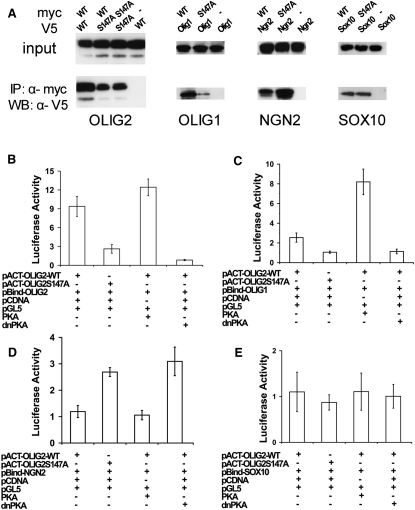
S147A Mutation Alters the Dimerization Properties of OLIG2 (A) Cos-7 cells were cotransfected with expression constructs encoding Myc-tagged OLIG2^WT^ or OLIG2^S147A^ together with V5-tagged OLIG2, OLIG1, NGN2, or SOX10. Cell lysates were immunoprecipitated with rabbit anti-Myc, followed by WB with anti-V5. In parallel, one-twentieth of each cell lysate was subjected directly to WB with anti-OLIG2. (B–E) Luciferase assay using cell lysates of transfected Cos-7 cells. The amounts of transfected DNA were normalized with pCDNA empty vector; *Renilla* Luciferase activity was used to calibrate transfection efficiency. Results are displayed as fold-increase of firefly Luciferase activity compared to controls (mean ± SEM of three independent experiments). See also Figure S2.

Given the location of the S147 phosphorylation site in the bHLH domain, it seemed likely that the phosphorylation status of this site might affect the interactions of OLIG2 with other proteins or with DNA. OLIG2 can form strong homodimers with itself as well as heterodimers with OLIG1 but forms weak heterodimers with other bHLH proteins, such as E12 or NGN2 (Lee et al., 2005; Li et al., 2007). It also interacts physically with other non-bHLH transcription factors, including SOX10, NKX2.2, and ZFP488 (Li et al., 2007; Sun et al., 2003; Wang et al., 2006; Wissmuller et al., 2006). We compared the cofactor-binding properties of OLIG2^S147A^ and OLIG2^WT^ by coIP assays in transfected Cos-7 cells and found that, compared to OLIG2^WT^, OLIG2^S147A^ had a reduced ability to form OLIG2-OLIG2 and OLIG2-OLIG1 dimers, whereas binding to NKX2.2, SOX10, or MASH1 was unaffected (Figures 2A and S2A). In contrast, OLIG2^S147A^ complexed more readily with NGN2 (Figure 2A). Together, these experiments indicate that S147A mutation does not destabilize OLIG2 or grossly affect its structure but, nevertheless, alters its binding to transcriptional partners.

We tried to mimic constitutive phosphorylation by mutating S147 to glutamic acid (E) or aspartic acid (D). However, both OLIG2^S147E^ and OLIG2^S147D^ exhibited reduced homodimer formation just like OLIG2^S147A^ (data not shown). This sort of effect is not unique to OLIG2. For example, phosphorylation/dephosphorylation of serine/threonine residues of the bHLH transcription factor HAND1 have been shown to regulate homodimer versus heterodimer formation, phosphorylation favoring heterodimers of HAND1 and E proteins and dephosphorylation favoring HAND1 homodimers (Firulli et al., 2003). However, mutation of the key serine/threonine phosphate acceptors to aspartic acid did not inhibit HAND1 homodimer formation, as would have been expected if these substitutions had mimicked constitutive phosphorylation, but instead strengthened homodimer formation just like serine/threonine → alanine substitution (Firulli et al., 2003).

We also compared the binding properties of OLIG2^WT^ and OLIG2^S147A^ using a mammalian two-hybrid system (CheckMate System, Promega) (Figures 2B–2E). This is a bipartite assay that depends on the physical association of test and target proteins at the promoter of a *Luciferase* reporter gene in Cos-7 cells, so activating Luciferase expression, which can be quantified by chemiluminescence. This assay confirmed the results of co-IP, namely, that association of OLIG2^WT^ with an OLIG2^S147A^ target was strongly reduced relative to either an OLIG2^WT^ or OLIG1 target (Figures 2B and 2C). In contrast, and also in agreement with the co-IP data, association of NGN2 with OLIG2^S147A^ was enhanced (Figure 2D). Furthermore, cotransfection of the catalytic subunit of PKA enhanced formation of OLIG2-OLIG2 and OLIG2-OLIG1 dimers while inhibiting OLIG2-NGN2 dimer formation, whereas a dnPKA had the opposite effect (Figures 2B–2E). In summary, phosphorylation of OLIG2 on S147, possibly by PKA, has a dramatic effect on cofactor choice, favoring NGN2 over other potential partners.

In addition we assessed the DNA binding activities of OLIG2^WT^ and OLIG2^S147A^ by electrophoretic mobility shift assay (EMSA). OLIG2^S147A^ exhibited significantly weaker binding to the HB9/M100 E box (Lee et al., 2005) compared to OLIG2^WT^ (Figure S2B). Given that dimerization is needed for bHLH proteins to bind to DNA targets (Murre et al., 1989), this result most likely reflects the reduced efficiency of OLIG2 homodimer formation as a result of S147A mutation.

### OLIG2^S147A^ Mutant Mice

**Figure 3:**
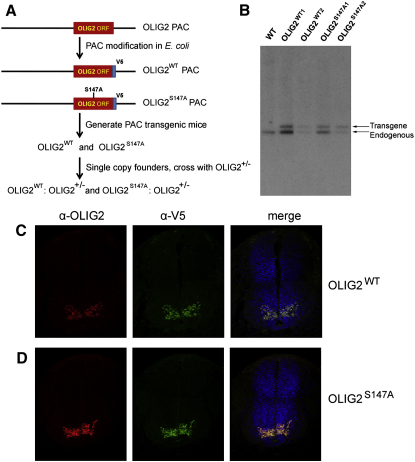
Construction and Characterization of OLIG2^S147A^ Transgenic Mice (A) The OLIG2 open reading frame in a mouse *Olig2* PAC was modified to contain a single mutation Serine147 → Alanine and a downstream V5-tag (*Olig2^S147A^*). A V5-tagged wild-type sequence was made as a control (*Olig2^WT^*). (B) Identification of single-copy transgenic founders by Southern blot. The transgene band was normalized to the endogenous *Olig2* band; founders whose transgene band was less intense than the endogenous band carried one copy of the *Olig2* transgene. (C and D) Transgenic embryos expressed OLIG2 faithfully in the ventral spinal cord. Transverse sections of E11.5 spinal cords from *Olig2^WT^* (C) and *Olig2^S147A^* (D) transgenic embryos were coimmunolabeled with OLIG2 (red) and the V5 epitope (green).

In order to study the phenotypic consequences of OLIG2-S147 phosphorylation in vivo, we generated *Olig2^S147A^* mutant mice. We modified OLIG2-coding sequence in an *Olig2* PAC clone (not containing *Olig1*), introducing the S147A mutation while simultaneously adding a V5 epitope tag to the C terminus (Figure 3A). Transgenic mice were generated by pronuclear injection. V5-tagged *Olig2^S147A^* and *Olig2^WT^* mice were made in parallel and single-copy founders of both lines were selected for further study (Figure 3B). Immunofluorescence microscopy confirmed that the *Olig2^S147A^* and *Olig2^WT^* PAC transgenes were faithfully expressed in the embryonic spinal cords of both lines (Figures 3C and 3D). We subsequently removed the endogenous *Olig2* alleles by crossing the PAC transgenes into an *Olig2* null background (Lu et al., 2002), thereby obtaining single-copy *Olig2^S147A^* and *Olig2^WT^* lines (i.e., *Olig2^S147A^*:*Olig2^−/−^* and *Olig2^WT^*:*Olig2^−/−^*). For some experiments we also bred the PAC transgenics with *Olig1/Olig2* double-null mice (Zhou and Anderson, 2002), which express green fluorescent protein (GFP) under transcriptional control of *Olig2* (see below).

### Olig2^S147A^ Mice Lack the pMN Precursor Domain in the Ventral VZ

**Figure 4:**
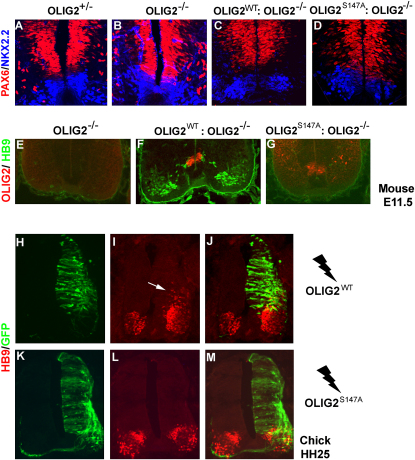
Ventral Patterning and MN Generation Are Disrupted in *Olig2^S147A^* Mice The pMN progenitor domain normally occupies the gap between PAX6 (red) and NKX2.2 (blue) immunolabeling in the ventral spinal cord of E11.5 embryos (A and C). pMN was missing in both *Olig2* null and *Olig2^S147A^* mice (B and D). MNs were immunolabeled with anti-HB9 (green) in the *Olig2^WT^* spinal cord (F) but were absent in *Olig2* null (E) and *Olig2^S147A^* (G). OLIG2^S147A^ protein (red) was still expressed in *Olig2^S147A^* mice (G). (H–M) *Olig2* expression constructs were electroporated in the HH12–14 chick neural tube. Electroporated cells were visualized 24 hr later by GFP immunolabeling (green). Ectopic HB9 expression (red) was induced by *Olig2^WT^* (H–J, white arrow), but not *Olig2^S147A^* (K–M). See also Figure S3.

Progenitors in p3, the ventral-most progenitor domain of the embryonic spinal cord, express the transcription factor NKX2.2, whereas progenitors in the p2 domain express IRX3 and a high level of PAX6 (Briscoe et al., 2000). The pMN domain lies between p3 and p2 and is marked by expression of OLIG2 and a low level of PAX6 (Lu et al., 2000; Zhou et al., 2000; Figure 4A). OLIG2 is essential for establishing and maintaining the pMN domain through cross-regulatory interactions with transcription factors in neighboring domains. For example, OLIG2 represses expression of *Irx3* and *Pax6* ; in the absence of OLIG2 function, *Irx3* and *Pax6* are derepressed in pMN, which takes on the character of p2, generating V2 interneurons and astrocytes instead of MNs and OLPs (Lu et al., 2002; Zhou and Anderson, 2002) (Figure 4B). This can be regarded as a “homeotic” transformation pMN → p2. We found that the pMN domain specifically was missing in our *Olig2^S147A^* mice (Figures 4C and 4D). Moreover, in *Olig2^S147A^:Olig2 ^GFP/−^*, *Olig1^+/−^* embryos, most pMN precursors (marked by GFP) were observed to adopt a p2 fate (high PAX6 expression) (Figure S3). These findings demonstrate that mutation of the S147 phosphate acceptor site destroys the neuroepithelial “patterning” function of OLIG2.

### Development of MNs Requires S147-Phosphorylated OLIG2

It is known that MNs fail to develop in the spinal cords of *Olig2^−/−^* embryos as a consequence of losing the pMN progenitor domain (Lu et al., 2002; Takebayashi et al., 2002; Zhou and Anderson, 2002; Figure 4E). Similarly, our O*lig2^S147A^* mutant mice failed to generate MNs, judging by the lack of expression of the MN-specific HD transcription factor HB9 (Figure 4G). HB9-positive MNs developed normally in *Olig2^WT^* mice (Figure 4F). It has been shown previously that forcing ectopic expression of *Olig2* in chick neural tube by electroporation at Hamilton-Hamburger (HH) stage12–14 can induce ectopic production of HB9-positive MNs by 48 hr post-electroporation, mainly in the p2 domain (Novitch et al., 2001; Mizuguchi et al., 2001; Lee et al., 2005). We confirmed this ability of *Olig2^WT^* to induce ectopic MNs (Figures 4H–4J) but found that *Olig2^S147A^* was inactive in this regard (Figures 4K–4M). Taken together, the data strongly suggest that S147 phosphorylation is necessary for the MN-inducing function of OLIG2.

### S147 Phosphorylation Is Dispensable for the OL-Inducing Activity of OLIG2

**Figure 5:**
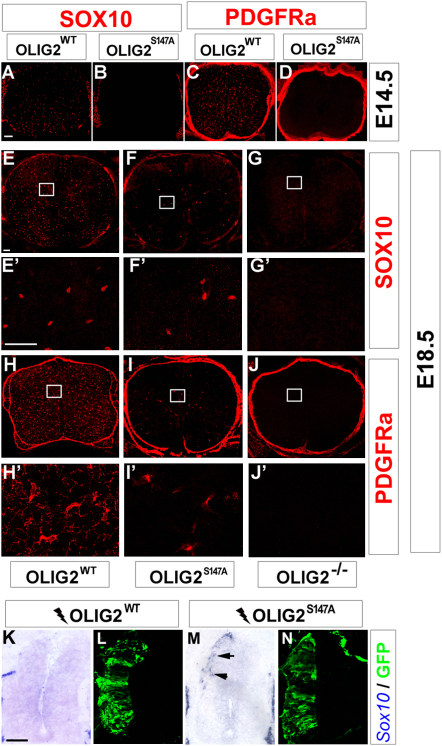
OL Specification in *Olig2^S147A^* and *Olig2^WT^* Mice OL lineage cells were marked by SOX10 or PDGFRa immunolabeling (red). OLPs were present in *Olig2^WT^* mice at E14.5 (A and C), but not in *Olig2^S147A^* mice (B and D). At E18.5, SOX10- and PDGFRa-positive OLPs were detected in spinal cords of *Olig2^S147A^* mice (F, F′, I, and I′), though in reduced numbers compared to *Olig2^WT^* controls (E, E′, H, and H′). No OLPs whatsoever were detected in *Olig2* null mice (G, G′, J, and J′). (E′–G′) and (H′–J′) are high-magnification views of the areas marked in (E)–(G) and (H)–(J), respectively. (K–N) *Olig2* expression constructs together with a GFP expression vector were electroporated into HH12–14 chick neural tubes, and electroporated cells were visualized 48 hr later by GFP immunolabeling (green). Ectopic *Sox10* expression (blue, arrows) was induced by transfected *Olig2^S147A^* (M and N), but not by *Olig2^WT^* (K and L). Scale bars, 80 μm (A–D); 50 μm (E–J′); 100 μm (K–N). See also Figure S4.

In the spinal cords of *Olig2* null mice, expression of the OLP markers PDGFRa and SOX10 is completely absent (Lu et al., 2002; Takebayashi et al., 2002; Zhou and Anderson, 2002), demonstrating an essential role for OLIG2 in OL lineage specification. In our *Olig2^S147A^* mutant mice, SOX10- and PDGFRa-expressing OLPs were missing at E14.5 (Figures 5A–5D) but appeared later at E18.5, though in reduced numbers (∼15%) relative to *Olig2^WT^* controls (Figures 5E, 5F, 5H, and 5I). At the time of their first appearance, OLPs were scattered through all regions of the cord, not concentrated in the ventral cord as in wild-type mice. This is consistent with the demonstrated loss of the ventral pMN domain (Figure 4)—the source of most but not all OLPs in the cord—and suggests that S147 phosphorylation is not required for OLP specification from other progenitor domains that do not rely on the prior neuroepithelial patterning function of OLIG2. We further investigated the effect of S147 phosphorylation on OLP differentiation into myelin-forming OLs by culturing primary E18.5 spinal cord cells under conditions permissive for OL differentiation. (It was not possible to study OL differentiation in vivo since *Olig2^S147A^* mutants die at birth due to the lack of MNs.) Myelin basic protein (MBP)-positive OLs formed in these mutant cell cultures as in wild-type cultures, demonstrating that S147 phosphorylation is not absolutely required for OL lineage progression (Figure S4).

To examine OLP-inducing activity further, we electroporated *Olig2^WT^* or *Olig2^S147A^* expression vectors into chick neural tube. It has been reported that *Olig2^WT^* can induce expression of OLP markers in the dorsal neural tube, after a delay of around 4 days post-electroporation (Liu et al., 2007). We found that forced expression of *Olig2^S147A^* induced dorsal SOX10 expression well ahead of this schedule at 48 hr post-electroporation (Figures 5M and 5N). As expected, *Olig2^WT^* did not induce SOX10 on this time scale (Figures 5K and 5L). Taken together, our data suggest that OL fate is favored, even accelerated, when OLIG2 is not phosphorylated on S147.

### OLIG2^S147A^ Favors OL Lineage Development in Cultured P19 Cells

**Figure 6:**
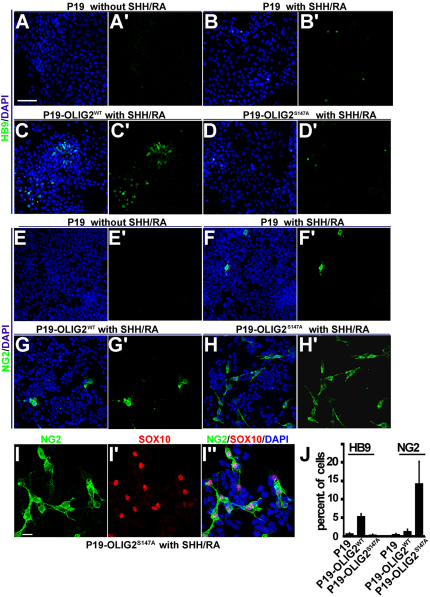
OLIG2^S147A^ Promotes OL Fate Specification but Represses MN Fate in Cultured P19 Cells (A–D) MN induction: addition of SHHAg1.2 agonist and RA induced aggregated P19 cells to express the MN marker HB9 (B and B′). This induction was accentuated in stable cell line P19-OLIG2^WT^ (C and C′) but repressed in P19-OLIG2^S147A^ (D and D′). (E–H) OL induction: SHHAg1.2 and RA also induced aggregated P19 cells to express OLP marker NG2 (F and F′). This induction was slightly amplified in the P19-OLIG2^WT^ line (G and G′) but dramatically magnified in P19-OLIG2^S147A^ (H and H′). (I, I′, and I″) The NG2-positive cells (green) that were induced in P19-OLIG2^S147A^ cultures also expressed SOX10 (red), confirming them as OL lineage cells. Cell nuclei were counterstained with DAPI (blue). (J) Quantification of HB9 and NG2-inducing activity. Scale bar, 50 μm (A–H′ and I–I″). See also Figure S5.

To investigate further the role of OLIG2-S147 phosphorylation in neural fate determination, we turned to an in vitro assay using P19 cells. This pluripotent embryonal carcinoma (EC)-derived cell line can differentiate into different cell lineages under appropriate inductive conditions (Jones-Villeneuve et al., 1982, 1983). For example, cell aggregation together with retinoic acid (RA) treatment drives the differentiation of P19 cells toward a neural fate, even as early as 4 hr postinduction (Berg and McBurney, 1990; Staines et al., 1996). We treated aggregated P19 cells with RA together with the SHH small-molecule agonist SHHAg1.2, a combination that can initiate MN development in cultured embryonic stem (ES) cells (Wichterle et al., 2002). We found that RA/SHHAg1.2 also induced the MN marker HB9 in aggregated P19 cells (Figures 6A and 6B). Furthermore, this treatment induced expression of proteoglycan NG2, which marks OLPs (Figures 6E and 6F).

To study the influence of OLIG2 on neural differentiation of P19 cells, we constructed two stable P19 lines that constitutively expressed V5-tagged OLIG2^WT^ or OLIG2^S147A^. Without RA/SHHAg1.2 induction, both cell lines grew and behaved like the parent P19 line. With RA/SHHAg1.2 induction, the P19-OLIG2^WT^ line produced significantly increased numbers of HB9-positive cells (p < 0.001) and NG2-positive cells (p < 0.05) compared to induced P19 control cells (Figures 6C and 6G). This is in keeping with previous reports that constitutive expression of OLIG2^WT^ can enhance the output of MNs and OL lineage cells from ES cells (Du et al., 2006; Shin et al., 2007). Under inducing conditions P19-OLIG2^S147A^ cultures developed a decreased number of HB9-positive cells (p < 0.05) compared with control P19 cells (Figure 6D), demonstrating a dominant-negative effect of the S147A mutant protein over endogenous, wild-type OLIG2 (which is also present in the P19 lines). Strikingly, P19-OLIG2^S147A^ cells induced with RA/SHHAg1.2 generated many more NG2-positive cells compared to induced P19-OLIG2^WT^ (p < 0.001) or parental P19 (p < 0.001) lines (Figures 6F–6J). The induced NG2-positive cells also expressed SOX10 (Figure 6I) and MBP (Figure S5). These data provide strong confirmation that loss of OLIG2-S147 phosphorylation directs NSCs away from an MN fate toward the OL lineage.

## Discussion

We have shown that phosphorylation of OLIG2 on S147 is required for the early functions of OLIG2 in neuroepithelial patterning and MN specification but is subsequently dispensable for OLP specification. We have also shown that S147 is phosphorylated during MN specification in the ventral spinal cord, dephosphorylated at the onset of OLP production, and that that dephosphorylation switches the binding preference of OLIG2 away from OLIG1/2 toward NGN2. We believe that this represents a key part of the regulatory mechanism that operates through OLIG2 to switch NSC fate from MNs to OLPs during ventral spinal cord development. Other phosphorylation events might also be important for the functional regulation of OLIG2, e.g., it was recently shown that casein kinase 2 (CK2)-mediated phosphorylation is required for the oligodendrogenic activity of OLIG2 (Huillard et al., 2010). Serine phosphorylation of NGN2 by glycogen synthase kinase-3 (GSK3) has also been shown to promote MN specification by facilitating assembly of NGN2 into a transcriptional complex with homeodomain factors LHX3 and ISL1/2 (Ma et al., 2008). It seems that phosphorylation of bHLH proteins (and perhaps other posttranslational modifications) might be a common means of regulating cell fate and lineage progression.

### OLIG2-S147 Phosphorylation and the MN-OL Fate Switch

Our data reveal that gain or loss of a phosphate group on OLIG2-S147 goes hand in hand with MN or OL generation, respectively. In our *Olig2^S147A^* mice, the pMN domain was transformed mainly to p2, and consequently, MN development was blocked. This does not reflect a global loss of OLIG2 function because expression studies in Cos-7 cells demonstrated that OLIG2^S147A^ is a stable protein that is indistinguishable from OLIG2^WT^ by mobility on sodium dodecyl sulfate (SDS)-PAGE, subcellular localization, or its ability to bind known transcriptional partners such as SOX10 or NKX2.2. Most importantly, OLIG2^S147A^ did not lose its ability to specify OL lineage cells, although fewer OLPs than normal developed in the spinal cords of *Olig2^S147A^* mice, and these were delayed, appearing at E15.5–17.5 instead of E12.5 as in wild-type cord. This fits with the fact that the pMN progenitor domain, which normally produces ∼80% of all OLPs in the cord, is lost in the mutant. The remaining ∼20% of OLPs are produced from more dorsal progenitor domains, which do not depend on the neuroepithelial patterning function of OLIG2 (Cai et al., 2005; Fogarty et al., 2005; Vallstedt et al., 2005). These dorsally derived OLPs are generated later than pMN-derived OLPs (∼E16.5 versus E12.5). They still require OLIG2 function for their development, for in *Olig2^−/−^* mice there are no spinal OLPs whatsoever (Lu et al., 2002; Takebayashi et al., 2002). It is very likely that the late-forming OLPs found in the *Olig2^S147A^* mutant correspond to these dorsally derived OLPs. The fact that they arise in the mutant demonstrates that the OLP-inducing function of OLIG2 is separable and distinct from its neuroepithelial patterning and MN-inducing functions. This conclusion is reinforced by the observation that *Olig2^S147A^* cannot induce ectopic MNs in chick electroporation experiments, yet can still induce the OL lineage marker *Sox10*. Moreover, *Olig2^S147A^* induces *Sox10* on an accelerated time course compared to *Olig2^WT^*, suggesting that *Olig2^S147A^* instructs NSCs to “leapfrog” MN production and go straight to OLPs. This separation between the MN- and OLP-inducing functions of OLIG2 was also strikingly confirmed by cell culture experiments; P19 cells (NSC-like) stably transfected with an *Olig2^S147A^* expression vector generated many more OL lineage cells—both NG2^+^ OLPs and MBP^+^ OLs—and less HB9^+^ MNs than did P19 cells stably transfected with *Olig2^WT^*. The MBP^+^ cells that formed in these experiments did not adopt the multiprocess-bearing morphology typical of normal OLs in culture, so other factors in addition to OLIG2 are presumably required for full OL differentiation of P19 cells.

We found that OLIG2^S147A^ has an enhanced ability to bind NGN2, coupled with a diminished ability to form dimers with itself or OLIG1. Consistent with this, we found that OLIG2^S147A^ inhibits NGN2-mediated transcriptional activation of the *HB9* promoter more efficiently than does OLIG2^WT^, in cotransfection assays with an HB9:luciferase reporter (Figure S6). NGN2 is a bHLH transcription factor that is known to be required for MN development because spinal MNs are not formed properly in mice lacking NGN2 (Scardigli et al., 2001). OLIG2 and NGN2 are coexpressed in the pMN domain and nowhere else, implying that OLIG2 and NGN2 act in concert during MN development (Mizuguchi et al., 2001; Novitch et al., 2001). There is evidence that OLIG2/NGN2 coexpression drives NSCs to exit the cell cycle and start expressing pan-neuronal markers (Mizuguchi et al., 2001; Novitch et al., 2001; Lee et al., 2005). NGN2 expression is later downregulated in pMN, and this was suggested to be necessary to enable pMN progenitors to switch from MN to OLP production (Zhou et al., 2001). However, in the light of our current data, we believe that NGN2 downregulation is not the trigger but rather a consequence of the MN-OLP switch that reinforces and stabilizes the gliogenic state.

**Figure 7:**
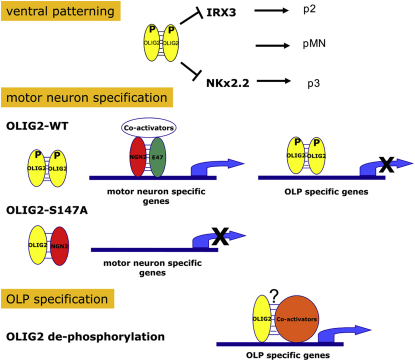
A Model of How OLIG2 Might Sequentially Regulate MN and OLP Fates through Reversible Phosphorylation on S147 A key feature is that dephosphorylation of OLIG2 increases the efficiency of OLIG2-NGN2 heterodimer formation, reducing the amount of NGN2 available for activating MN-specific genes and thereby contributing to the neuron-OL fate switch.

Taken together with previous research, our data provide new ideas about the chain of events leading up to and beyond the MN-OLP fate switch. We propose that during the early neurogenic phase (∼E9–E12), homodimers of S147-phosphorylated OLIG2 act to repress OL lineage genes in pMN and create a permissive environment for MN development—in which NGN2 plays an important role in concert with homeodomain transcription factors ISL1/2 and LHX3 (Lee and Pfaff, 2003; Lee et al., 2005; Ma et al., 2008). Subsequently, dephosphorylation of OLIG2-S147 disrupts OLIG2 homodimers and encourages formation of heterodimers such as OLIG2/NGN2, thereby sequestering NGN2 and possibly other bHLH factors and shutting down MN lineage genes. At the same time, OLIG2 associates with other unidentified cofactors to activate the OL genetic program and repress the MN program (including NGN2), hence reinforcing the neuron-glial switch. This scheme is illustrated in Figure 7. However, we note that endogenous MN development did not appear to be inhibited in OLIG2^S147A^:OLIG2^+/−^ mice (data not shown) or in OLIG2^S147A^-electroporated chick (Figure 4L), which could be taken to argue against the simple sequestration model depicted. However, it is possible that in both these situations OLIG2^S147A^ expression might not have been robust enough to completely overcome endogenous OLIG2 function.

A potential partner of OLIG2 that might come into play during OL lineage specification is NKX2.2. Forced expression of OLIG2 together with NKX2.2 in chick neural tube induces early onset of OLP specification (Sun et al., 2001). In addition, OLIG2 and NKX2.2 can physically interact with each other (Sun et al., 2003). However, OLIG2 and NKX2.2 are not tightly colocalized in the pMN domain in the run up to MN-OLP fate switching in mice (Sun et al., 1998; Richardson et al., 2006), so it is not clear that OLIG2 and NKX2.2 normally work together in OLP specification. In fact, OLPs develop normally in NKX2.2 null mice, although their subsequent differentiation into OLs is prevented (Qi et al., 2001). Another potential cofactor for OLIG2 is MASH1/ASCL1; initially (∼E12.5) there are reduced numbers of OLPs in MASH1 null spinal cords, but this soon resolves, suggesting that MASH1 is not critical for OLP specification (Sugimori et al., 2008). We found that S147A mutation did not have a noticeable effect on binding of OLIG2 to either NKX2.2 or MASH1 (Figure S2A), so we probably have to look elsewhere for critical determinants of OL lineage specification in the spinal cord. The transcription factor NF1A has been proposed to trigger gliogenesis throughout the neural tube (Deneen et al., 2006), but its physical and functional relationships with OLIG2 have yet to be investigated. Recently, OLIG2 was shown to interact with the bHLH protein E47 to directly activate the *Sox10-U2* promoter in the OL lineage (Küspert et al., 2010). The results of our present study suggest that dephosphorylated OLIG2^S147^ might specifically interact with E47 in this context.

Epigenetic modifications play an important part in cell fate decisions, including OLP specification. For example, in the absence of histone deacetylases 1 and/or 2 (HDAC1/2), OLP formation in the ventral spinal cord is disrupted (Cunliffe and Casaccia-Bonnefil, 2006; Ye et al., 2009; Li et al., 2009). It will be important in the future to investigate the potential interactions between OLIG2, HDACs, and other histone modifiers (e.g., methylases) and how these interactions might be influenced by posttranslational modification.

### The Role of PKA

Our study focused on reversible phosphorylation of OLIG2-S147, an evolutionarily conserved site that is predicted to be a PKA target. We provided evidence that OLIG2-S147 can be phosphorylated by PKA in cultured Cos-7 cells and that dnPKA inhibits phosphorylation. Despite this, we cannot conclude that PKA is the only—or even the primary—protein serine kinase to target S147 in vivo. For example, the consensus target sequence for PKA (R[R/K]X[S/T]) can overlap with that of PKC ([R/K]X[S/T]U[R/K]) (U, hydrophobic). Indeed, PKA and PKC are thought to phosphorylate common acceptor sites on the bHLH proteins HAND1 and HAND2 (Firulli et al., 2003).

Nevertheless, the fact that *Olig2^S147A^* mice failed to develop MNs suggests that PKA might normally play a positive role in MN development. This seems counter to the popular idea that PKA negatively regulates the SHH pathway (Li et al., 1995; Ruiz i Altaba, 1999; Epstein et al., 1996; Hammerschmidt et al., 1996; Jacob and Briscoe, 2003). SHH signaling works through the transcription factors GLI1–3, vertebrate homologs of *Drosophila* Cubitus interruptus (Ci). SHH activates GLI3 (and GLI2) by inducing its proteolytic conversion from a full-length transcriptional activator into a truncated N-terminal repressor (Ruppert et al., 1990; Dai et al., 1999; Wang et al., 2000). It is believed that PKA phosphorylation stimulates GLI3 cleavage (Wang et al., 2000; Tempe et al., 2006), which might underlie the repressive action of PKA on SHH signaling since truncated GLI3 represses the SHH pathway (Dai et al., 1999; Wang et al., 2000; Bai et al., 2004; Tempe et al., 2006). GLI3 is not the only component of the SHH pathway that can be PKA phosphorylated, and PKA has been shown to play a positive as well as a negative role in SHH signaling. For example, in *Drosophila*, PKA phosphorylation of SMO, the Hedgehog (HH) coreceptor, promotes SMO accumulation on the primary cilium and triggers HH pathway activation (Jia et al., 2004). Also, during limb development, elevating PKA activity by Forskolin treatment or by infecting with a retroviral PKA expression vector exerts a positive effect on SHH signaling, resulting in an altered pattern of digits (Tiecke et al., 2007).

We examined the effect of stimulating the PKA pathway in neonatal mouse cortical cell cultures with Forskolin and dibutyryl cyclic AMP (db-cAMP), a cell-permeable analog of cAMP, and found that the number of NG2-positive OLPs was significantly decreased compared to untreated cultures (Figure S7). This is consistent with expectation since our other data predict that elevating PKA activity should increase OLIG2-S147 phosphorylation and stimulate neurogenesis at the expense of OLPs. However, in the light of the above discussion, it is clear that PKA probably has multiple parallel functions, and our experiments with Forskolin/db-cAMP should be interpreted cautiously.

### What Signals S147 Dephosphorylation?

Our data raise the obvious question: What is the key event that signals S147 dephosphorylation and triggers the MN-OLP switch in pMN? Notch signaling is known to play an important role in glial cell development in the CNS (Wang et al., 1998; Park and Appel, 2003; Park et al., 2005; Deneen et al., 2006). Constitutive activation of components of the Notch pathway in chick spinal cord can downregulate NGN2 expression in pMN and initiate OLP generation (Zhou et al., 2001). Notch1 is expressed by neuroepithelial cells throughout the neural tube, and its ligand Jagged-2 is expressed exclusively in the pMN domain of zebrafish spinal cord during late neurogenesis (Yeo and Chitnis, 2007). These and other observations frame the Notch pathway as a potential key player in the MN-OLP switch. It is possible that activated Notch-1, via its effector HES5, might induce expression of specific phosphatases and/or repress phosphatase inhibitors, resulting in dephosphorylation of OLIG2-S147 and initiation of OLP production. It will be worth exploring these ideas in the future. By improving our understanding of OL lineage specification, this work might aid the design of procedures for efficient large-scale production of OL lineage cells for clinical use—cell replacement therapy for demyelinating diseases like multiple sclerosis, for example—or for high-throughput drug screens. Study of the posttranslational pathways that modulate transcriptional activity of OLIG2, NGN2 and other related factors might also provide insights into general mechanisms of stem cell fate choice.

## Experimental Procedures

### Plasmid Vectors

Open reading frames of mouse *Olig1*, *Olig2*, *Sox10*, *Ngn2*, *Mash1*, and *Nkx2.2* were amplified by RT-PCR from RNA extracted from mouse embryonic spinal cord tissue and subcloned into Invitrogen's pCDNA3.1-V5 and/or pCDNA4-myc vectors, or Promega's pBIND and/or pACT vectors (for two-hybrid assays). Catalytic PKA and dnPKA vectors were kindly provided by Dr. Judy Varner (University of California, San Diego). S147A mutant vectors were generated using Stratagene's QuikChange Site-Directed Mutagenesis Kit following the manufacturer's instructions. The HB9- luciferase reporter plasmid HB9:luc (Lee et al., 2005) was a gift from Dr. Samuel Pfaff (Salk Institute, La Jolla, CA, USA).

### Antibodies and In Situ Hybridization Probes

Primary antibodies used for WB and IP were as follows: goat anti-OLIG2 (R&D Systems; used at 1:250 dilution for IP); rabbit anti-OLIG2 (a gift from Charles Stiles, Dana Farber Cancer Institute; 1:20,000 for WB); mouse anti-Myc (Sigma; 1:5,000 for WB); rabbit anti-Myc (Abcam; 1:500 for IP); and mouse anti-V5 (Abcam; 1:2,500 for WB). The specific anti-OLIG2 ^ph-S147^ antibody was generated by CovalAb UK, Cambridge. The sequence of the immunizing peptide was YAHGPSVRKL-(phospho-S147)-KIA (residues 137–150 of OLIG2). Rabbits were immunized four times with peptide, and antiserum was collected 25 days after the last injection. The antiserum was first absorbed on a column containing nonphosphorylated peptide and then affinity purified on a phosphopeptide column. The purified antibody was used in WB at a dilution of 1:250. Primary antibodies used for immunofluorescence labeling were: goat anti-OLIG2 (R&D Systems; 1:750); rabbit anti-OLIG2 (from Charles Stiles; 1:4000); chick anti-GFP (Aveslab; 1:1000); rabbit anti-V5 (Abcam; 1:250); rabbit anti-Pax6 (Chemicon; 1:500); mouse anti-Nkx2.2 (Developmental Studies Hybridoma Bank [DSHB] supernatant; 1:50); mouse anti-HB9 (DSHB; 1:20); guinea pig anti-SOX10 (a gift from Michael Wegner, Erlanger University, Germany; 1:2000); and rat anti-PDGFRa (BD PharMingen; 1:400). Secondary antibodies for WB were bought from Thermo Pierce and used at 1:20,000. Alexa Fluor secondary antibodies were from Invitrogen (used at 1:750 dilution). The in situ hybridization probes for mouse *Sox10* and *Pdgfra* were described previously (Tekki-Kessaris et al., 2001). The chick *Sox10* probe was generated by in vitro transcription from a plasmid containing a chick *Sox10* genomic fragment.

### Mouse Lines

The *Olig2* KO line was obtained from Charles Stiles (Dana Farber Cancer Institute, Harvard Medical School) and David Rowitch (University of California, San Francisco) (Lu et al., 2002). The *Olig1/Olig2* double-null line (*Olig1^−/−^, Olig2^GFP/GFP^*) was provided by David Anderson (California Institute of Technology) (Zhou and Anderson, 2002). To create *Olig2^WT^* and *Olig2^S147A^* transgenic lines, a mouse PAC clone containing a ∼200 kb *Olig2* genomic fragment was modified by homologous recombination in *E*. *coli* (Lee et al., 2001). The 5′-homology fragments were subcloned into *pCDNA3.1-Olig2^WT^-V5* or *pCDNA3.1-Olig2^S147A^-V5*. The modified PAC constructs were linearized with PvuI and purified by pulsed field gel electrophoresis for pronuclear injection. Transgenic founders were screened by Southern blot of BglII-digested genomic DNA, and single-copy founders were selected to establish lines. The radiolabeled probe for Southern blotting detected a sequence in the 3′UTR of the *Olig2* gene. Progeny of transgenic founders were crossed first with *Olig2^+/−^* mice (Lu et al., 2002) to obtain *Olig2^S147A^:Olig2^+/−^* and *Olig2^WT^:Olig2^+/−^* offspring of both sexes, which were then sibling mated to obtain *Olig2^S147A^* (i.e., *Olig2^S147A^:Olig2^−/−^*) and *Olig2^WT^* (*Olig2^WT^:Olig2^−/−^*) offspring for analysis. For certain experiments (e.g., Figure S3), we crossed *Olig2^S147A^*:*Olig2^+/−^* or *Olig2^WT^*:*Olig2^+/−^* animals to *Olig1/2* double knockouts, which express GFP under control of the *Olig2* locus (Zhou and Anderson, 2002), to obtain *Olig2^S147A^:Olig2 ^GFP/−^*, *Olig1^+/−^*, and *Olig2^WT^:Olig2 ^GFP/−^*, *Olig1^+/−^* embryos.

### Cell Culture

Cos-7 cells were cultured in Dulbecco's modified Eagle's medium (DMEM) supplemented with 10% (v/v) fetal bovine serum (Invitrogen) at 37°C with 5% (v/v) CO_2_. Plasmid transfection was performed using Lipofectamine 2000 reagent (Invitrogen). Total DNA concentrations were normalized with empty vector DNA where required. The P19 EC-derived cell line was purchased from LGC-ATCC and maintained in Alpha minimal essential medium with ribonucleosides and deoxyribonucleosides, supplemented with 5% fetal bovine serum (Invitrogen) at 37°C with 5% CO_2_. For differentiation assays, 5 × 10^4^ P19 cells were plated on 35 mm diameter plates in medium with 1% serum. After 5–6 days, the aggregated cells were treated with 1 μM RA and 100 nM SHHAg1.2 (Curis, Inc.). Cells were fixed for immunolabeling after two more days in culture.

### IP and WBs

Cultured cells were lysed by homogenization in 50 mM Tris-HCl (pH 7.5), 150 mM NaCl, 1% (v/v) Nonidet P-40, 0.5% (w/v) sodium deoxycholate, and one tablet of protease inhibitor mix per 50 ml buffer. Lysis buffer for spinal cord tissue was purchased from Sigma (CelLytic MT); sometimes phosphatase inhibitors were included. After lysis, cell debris was removed by centrifugation at 30,000 × g, then 500 μl cell lysate was treated with 50 U of DNase1 and precleared with 30 μl of protein G beads (GE Healthcare) for 3 hr at 4°C on a rotating wheel. The supernatant was decanted, incubated with the antibody of interest at 4°C for 1 hr, mixed with 30 μl of protein G beads, and incubated overnight at 4°C. The beads were washed twice in lysis buffer, twice in high-salt buffer (50 mM Tris-HCl [pH 7.5], 500 mM NaCl, 0.1% [v/v] Nonidet P-40, 0.05% [w/v] sodium deoxycholate) and once in low-salt buffer (50 mM Tris-HCl [pH 7.5], 0.1% Nonidet P-40, 0.05% sodium deoxycholate). The beads were resuspended in 30 μl of SDS gel sample buffer, boiled for 5 min, and subjected to SDS-PAGE followed by WB. Band intensity was quantified using ImageJ software. For 2D PAGE the beads were treated with Invitrogen ZOOM Protein Solubilizer, and protein samples were separated on the Invitrogen ZOOM 2D gel system following the manufacturer's instructions. After electrophoresis, proteins were transferred to polyvinylidene difluoride membranes. Protein bands were visualized by chemiluminescence (ECLplus kit; GE Healthcare).

### In Vitro Phosphorylation Assays

Thirty-six hours after transfection, Cos-7 cells were washed twice with phosphate-free DMEM (Invitrogen). Thirty mega-becquerels [^33^P]orthophosphate (Amersham) were added to phosphate-free medium, and cells incubated for 4 hr at 37°C. The medium was then removed, and the cells washed twice with ice-cold PBS and immediately lysed on ice (see above). Proteins were immunoprecipitated, separated by SDS-PAGE, and visualized by autoradiography.

### Two-Hybrid Protein Binding Assays

Two-hybrid assays for protein-protein interactions were performed using Dual Luciferase Assay System (Promega). The amounts of transfected DNAs were normalized with empty pCDNA vector. The measured firefly luciferase activity was normalized against *Renilla* Luciferase activity. Three independent transfections were conducted in parallel for each condition, and each experiment was repeated three times.

### In Ovo Electroporation of Chick Embryos

Fertilized chicken eggs were supplied by Henry Stuart Inc. and incubated at 38°C in a humidified atmosphere. The embryos were staged according to HH and electroporated at HH12–14 (Hamburger and Hamilton, 1992). Expression constructs were diluted in injection buffer (3 μg/μl in PBS containing 0.8% [w/v] Fast Green), injected into the spinal cord lumen, and electroporated using an Intracel TSS20 Ovodyne electroporator with EP21 current amplifier. Embryos were analyzed 48 hr later.

### Immunohistochemistry and In Situ Hybridization

Mouse and chick embryos were dissected in cold PBS and fixed in 4% (w/v) paraformaldehyde (PFA) in PBS for 1 hr at 20°C or overnight at 4°C (for SOX10 immunolabeling). The tissues were cryoprotected with 20% (w/v) sucrose in PBS, embedded in OCT, and frozen for cryosectioning. Tissue sections (15 μm) were permeabilized and preblocked in 0.1% (v/v) Triton X-100, 2% (v/v) calf serum in PBS for 1 hr at 20°C, then incubated in primary antibodies diluted in 2% calf serum in PBS overnight at 4°C followed by secondary antibodies at 20°C for 1 hr. Sections were counterstained with Hoescht 33258 (1:1000; Sigma) to visualize cell nuclei. For RNA in situ hybridization, see http://www.ucl.ac.uk/∼ucbzwdr/Richardson.htm.

## Supplemental Information

Document S1. Seven Figures, One Table, and Supplemental Experimental Procedures
