# Supplementary material for: Phosphorylation Regulates OLIG2 Cofactor Choice and the Motor Neuron-Oligodendrocyte Fate Switch
Source: Neuron. 2011 Mar 10;69(5):918–29. doi: 10.1016/j.neuron.2011.01.030 (PMC3093612; doi:10.1016/j.neuron.2011.01.030)
Supplement: Document S1. Seven Figures, One Table, and Supplemental Experimental Procedures [file mmc1.pdf]

Neuron, Volume *69*

Supplemental Information

Phosphorylation Regulates OLIG2 Cofactor Choice

and the Motor Neuron-Oligodendrocyte Fate Switch

Huiliang Li, Joana Paes de Faria, Paul Andrew, Justyna Nitarska, and William D. Richardson

Inventory of Supplemental Information

1. Supplemental Experimental Procedures

2. Supplemental Tables

Table S1, related to Fig. 1

3. Supplemental Figures

Figure S1, related to Fig. 1

Figure S2, related to Fig. 2

Figure S3, related to Fig. 4

Figure S4, related to Fig. 5

Figure S5, related to Fig. 6

Figures S6 and S7, related to Discussion

4. Supplemental References

Supplemental Information

Supplemental Experimental Procedures:

Primary cell culture

For neocortical cell culture, E13.5 cerebral hemispheres were dissected in Earles Buffered Saline Solution(EBSS) without Ca^{2+} or Mg^{2+} and incubated in EBSS with dispase (1 mg/ml) and DNase 1 for ~10 minutes at room temperature (20-23°C), then the ganglionic eminences were removed with forceps. Isolated cortices were digested in 2 ml of EBSS with 0.01% (w/v) Trypsin and DNase1 for 45 minutes at 37°C. 3×10^5 cells were plated in 350 μl defined medium ("Sato's medium"; Bottenstein and Sato., 1979) in each well of poly-D-lysine coated 24-well plates and cultured at 37°C in a 5% CO_2 atmosphere. Cells were treated with 10 ng/ml FGF2 and different concentrations of the PKA activators forskolin or dibutyryl-cAMP. For OL differentiation culture, E18.5 thoracic spinal cord was dissected and cells dissociated as above. The cells were cultured in Sato's medium with 20 ng/ml FGF2 for three days, then the cells were washed with DMEM and cultured for a further six days in Sato's medium without FGF2 before immunolabelling for MBP.

Electrophoretic mobility shift assay (EMSA)

^{32}P-labelled, double-stranded oligonucleotide (CAGCCTTTCAGTGGAATTTCCCAGATGGGCCAA-GGGGAGGCCAGG) containing the HB9 M100 E-box (EB) site (Lee et al., 2005) was used as probe (transcription factor binding site is underlined). OLIG2 protein was translated in vitro from pCDNA-OLIG2-myc vector using the T7 TNT system (Promega) following the manufacturer's instructions. 20 μl of in vitro translation product was incubated at 20-23°C for 10 minutes in reaction buffer [20% (v/v) glycerol, 20 mM HEPES, 100 mM KCl, 0.2 mM EDTA, 0.5 mM DTT, 0.01 Unit poly(dI-dC) and 25 ng/μl sheared salmon sperm DNA]. A 1 μl aliquot of protein was subjected to Western blot and the rest of the protein solution was incubated with 1 μl of 0.1 pmol radiolabeled probe for 20 min. For competitive binding experiments, 10 pmol of unlabeled probe was added along with the radiolabeled probe. For supershift experiments, 1 μg of mouse anti-Myc antibody was added. All reaction mixtures were run simultaneously on a 6% (w/v) polyacrylamide non-denaturing Tris-borate EDTA Novex minigel (Invitrogen) until the dye had migrated off the end of the gel. The gel was dried and visualized by autoradiography.

Table S1. Properties of mouse OLIG2 protein

Amino Acid composition	323 aa (50Ser, 14Thr, 3Tyr)
bHLH domain	110-167aa
Potential PKA site	S147
Potential PKC site	S9, T38, S81,T91, S93, T94, S253
Predicted serine phosphorylation sites	S6, S9, S13, S14, S26, S30, S31, S42, S45, S78, S80, S81, S83, S84, S85, S87, S92, S93, S115, S147, S165, S174, S250
Predicted threonine phosphorylation sites	T94, T319
Tyrosine phosphorylation sites	None

Potential phosphorylation sites of mouse OLIG2 were predicted by NetPhosK 1.0 Server (http://www.cbs.dtu.dk/services/NetPhosK/), with 0.75 threshold cut-off. Predictions for Serine, Threonine and Tyrosine phosphorylation sites were produced by NetPhos 2.0 server (http://www.cbs.dtu.dk/services/NetPhos/).

SUPPLEMENTAL FIGURES

Figure S1. ELISA assay of OLIG2 $^{ph\text{-}S147}$ phosphorylation-specific antibody.

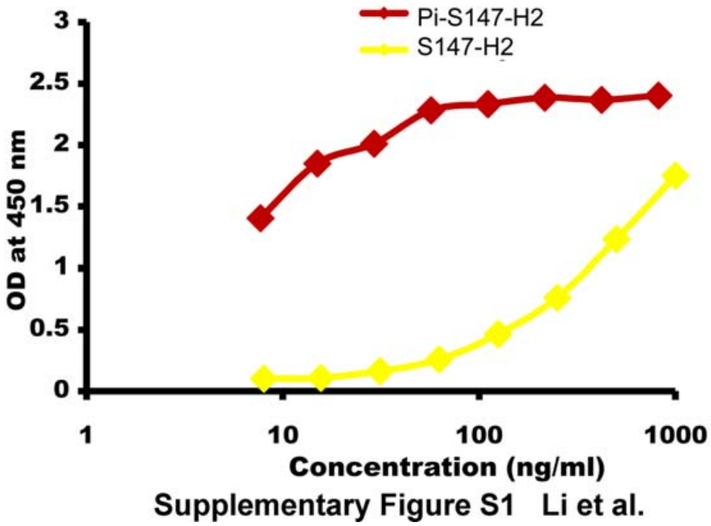

OLIG2 peptide (residues 137 to150), either phosphorylated on S147 (red) or not (yellow), were coated onto 96-well microtiter plates. Purified anti-OLIG2$^{ph\text{-}S147}$ antibody was applied at a range of concentrations and optical density (OD) was read at 450 nm.

Figure S2. A. S147A mutation did not alter OLIG2 binding to MASH1 or NKX2.2.

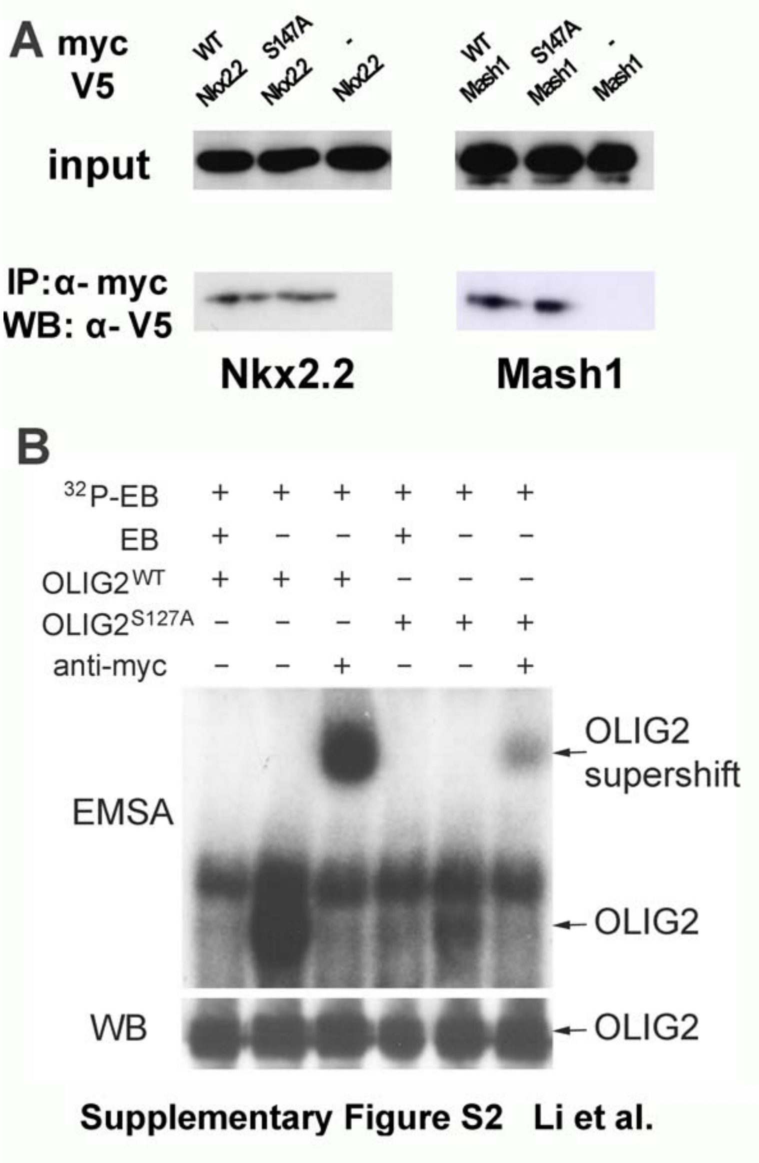

Myc-tagged $OLIG2^{WT}$ or $OLIG2^{S147A}$ together with V5-tagged MASH1 or NKX2.2 were co-transfected into cultured Cos-7 cells. Cell lysates were immuno-precipitated with rabbit anti-Myc antibody, followed by Western blot with mouse anti-V5 antibody. One-twentieth of the each cell lysate was directly subjected to Western blot with anti-OLIG2. **B.** Analysis of OLIG2 DNA binding activity by EMSA. OLIG2 could shift a ^{32}P-labeled 45 bp double-stranded oligonucleotide containing the HB9 M100 E-box (EB) site (Lee et al., 2005). 50-fold excess of unlabeled oligonucleotide was an effective competitor. Mouse anti-Myc IgG was used for supershift. The S147A mutant displayed decreased DNA binding activity compared to wild type OLIG2. Western blot indicated uniform protein loading.

Figure S3.

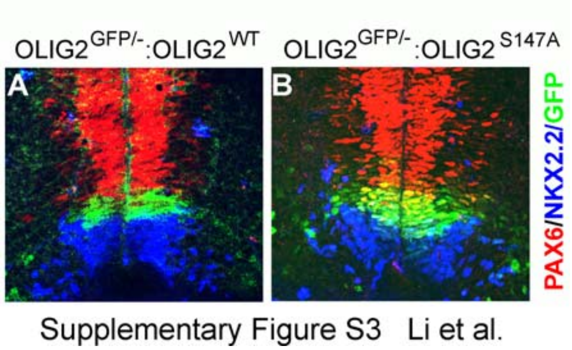

(**A, B**) pMN progenitors are marked by GFP under *Olig2* transcriptional control in *Olig2*$^{GFP/-}$: *Olig1*$^{+/-}$ mice (Zhou and Anderson, 2002). *Olig2*^{WT} or *Olig2*^{S147A} PAC transgenes were crossed into this genetic background to allow *Olig2*-expressing progenitor cells to be visualized in sections by GFP immunolabelling (see Methods for details of genotypes and breeding strategy). GFP expression filled the gap between the p2 progenitor domain (high PAX6 expression, red) and the p3 domain (NKX2.2 expression, blue) in spinal cords of *Olig2*^{WT} mice (A), contrasting with *Olig2*^{S147A} mutant mice in which the pMN domain (PAX6-low, NKX2.2-negative) was lost and GFP-positive cells were re-specified mainly as p2 progenitors (PAX6-high) (B).

Figure S4. OL differentiation is unaffected by OLIG2^{S147A} mutation.

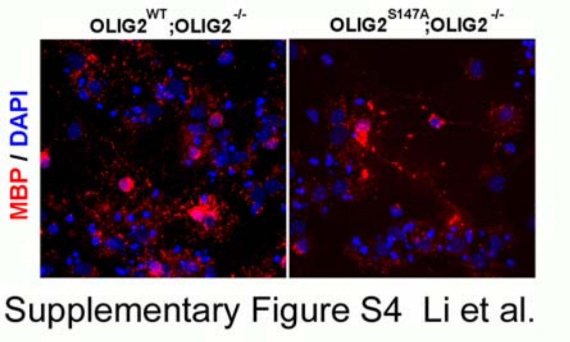

(**A**) E18.5 mouse spinal cords were isolated and dissociated cells cultured in Sato's defined medium for three days with 20 ng/ml FGF2 followed by five days without FGF2. The cells were then fixed and immunolabelled with anti-MBP antibody. Scale bar: 20 μm.

Figure S5. P19-OLIG2^{S147A} and P19-OLIG2^{WT} cells were treated with retinoic acid (RA) and the Sonic hedgehog agonist SHHAg1.2.

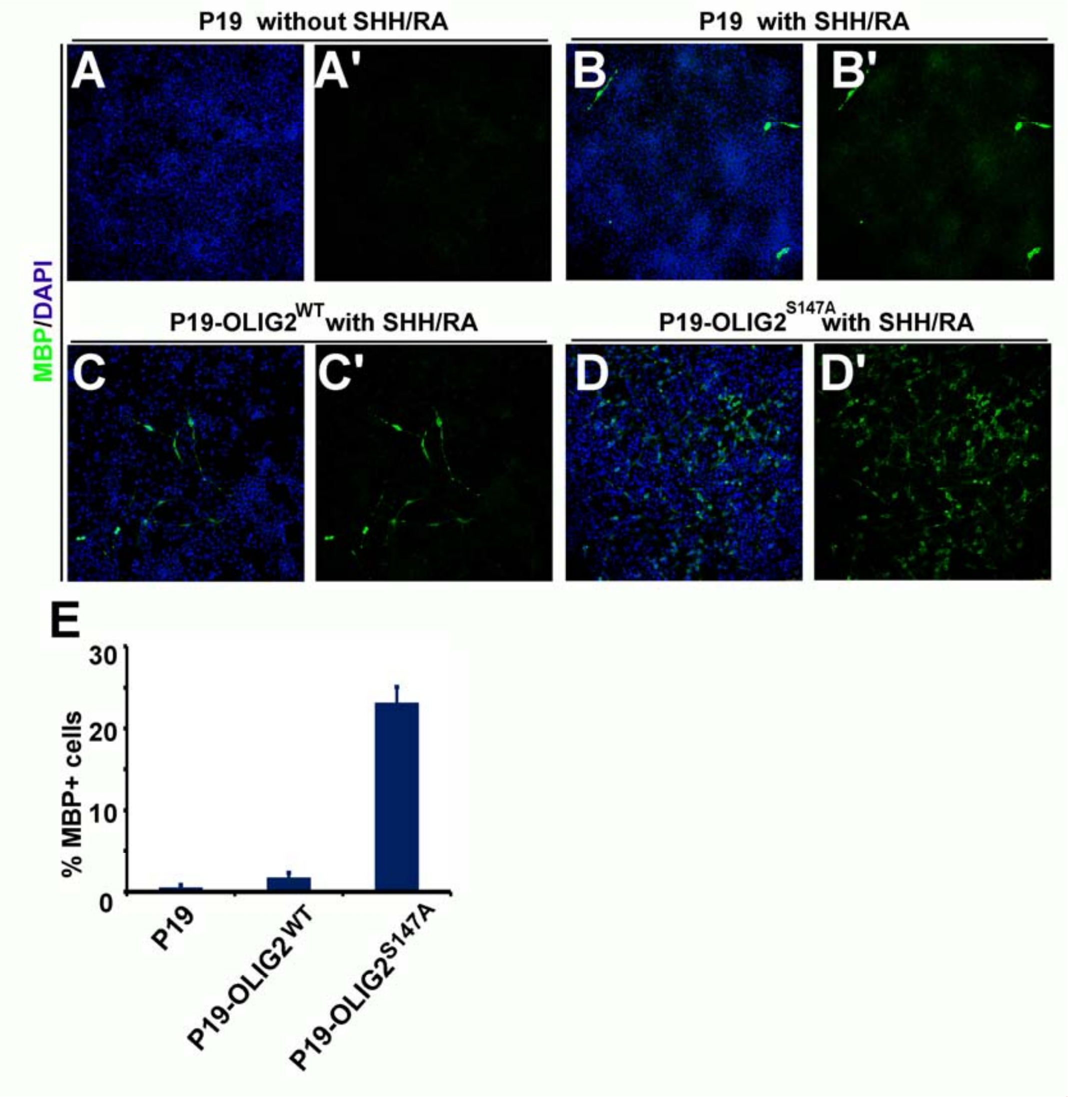

Supplementary Figure S5 Li et al.

The OLIG2^{S147A} mutation strongly enhanced expression of the differentiation marker MBP. However, morphological differentiation (formation of multi-process bearing cells) was not observed over the time course of the experiment.

Figure S6. NGN2-mediated transcription of a luciferase reporter from the HB9 promoter was repressed by OLIG2.

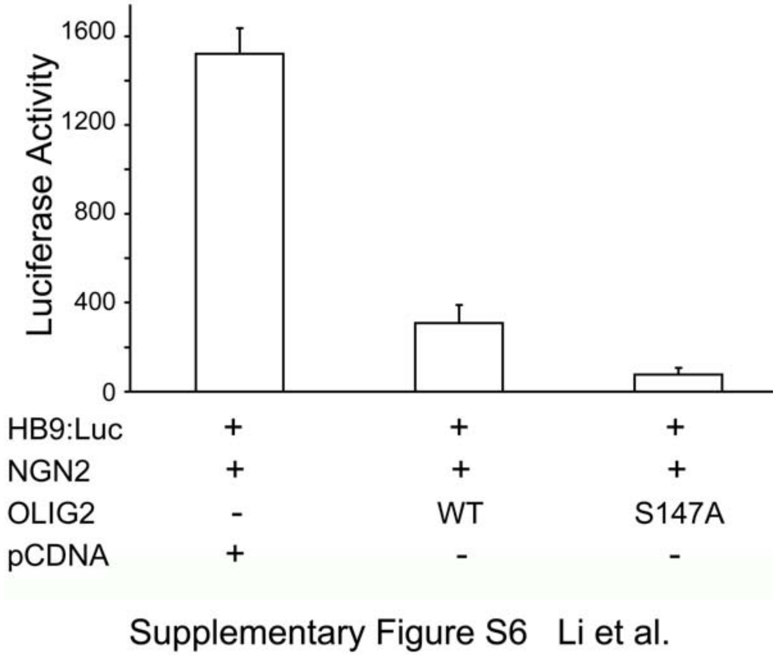

The luciferase assay was performed with cell lysates from transfected P19 cells. pCDNA empty vector was used to normalize the amounts of transfected DNA. Results are the mean ± s.e.m. of three independent experiments. OLIG2^{S147A} displayed increased repressive activity compared to OLIG2^{WT}.

Figure S7. Dissociated cells from mouse E13.5 neocortex were cultured in defined medium in the presence of 10 ng/ml FGF2 for three days.

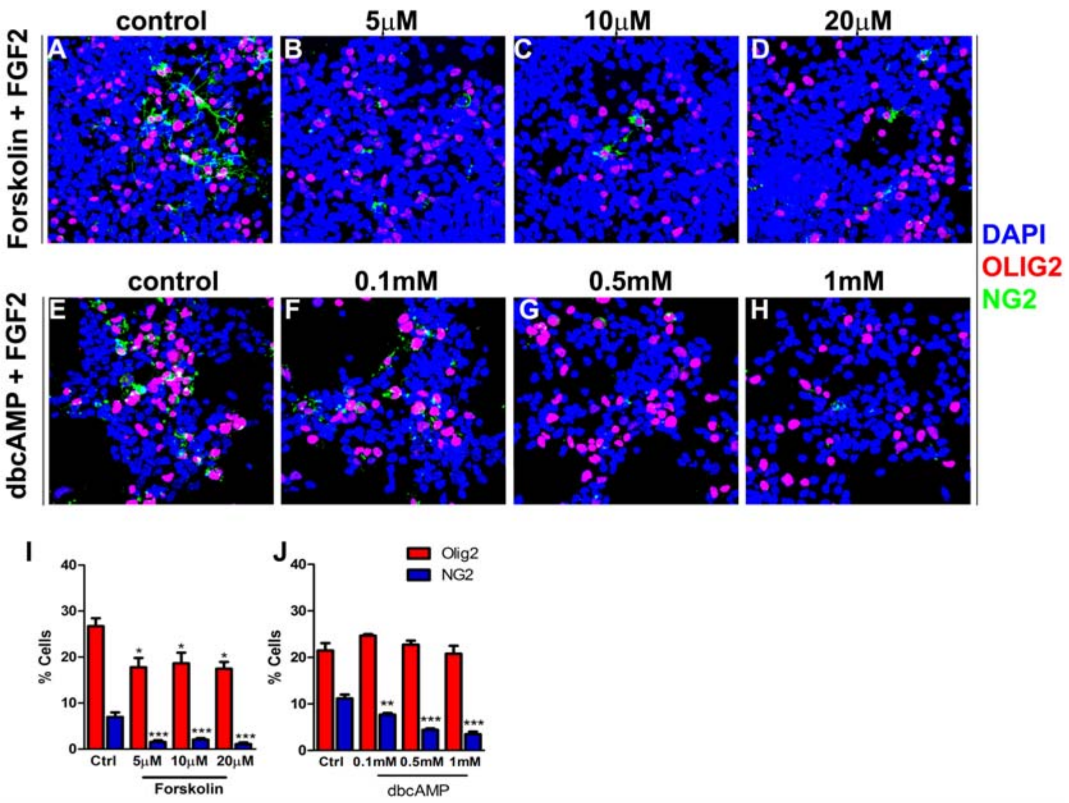

Supplementary Figure S7 Li et al.

Various concentrations of PKA activators forskolin (A-D) or dibutyryl-cAMP (E-H) were added. Cells were fixed and immuno-labelled with anti-NG2 (green) and anti-OLIG2 (red). Cells in two randomly selected fields on each of three microscope slides were counted under the 40X objective. The proportions of labelled cells were plotted as mean ± s.e.m. (I, J). Statistical comparisons were by one-way ANOVA (* = $p<0.05$; ** = $p<0.01$; *** = $p<0.001$). The results shown here were from one of at least two independent experiments, all of which gave similar results.

Supplemental References

Bottenstein,J.E., and Sato,G.H. (1979). Growth of a rat neuroblastoma cell line in serum-free supplemented medium. Proc. Natl. Acad. Sci. USA *76*, 514-517.

Lee,S.K., Lee,B., Ruiz,E.C., and Pfaff,S.L. (2005). Olig2 and Ngn2 function in opposition to modulate gene expression in motor neuron progenitor cells. Genes Dev. *19*, 282-294.
